# “An unnecessary cut?” multilevel health systems analysis of drivers of caesarean sections rates in Italy: a systematic review

**DOI:** 10.1186/s12884-020-03462-1

**Published:** 2020-12-10

**Authors:** Valentina Laurita Longo, Emmanuel Nene Odjidja, Thierry Kamba Beia, Manuela Neri, Karina Kielmann, Irene Gittardi, Amanda Isabella Di Rosa, Michela Boldrini, Gian Benedetto Melis, Giovanni Scambia, Antonio Lanzone

**Affiliations:** 1 Department of Surgical Sciences, Department of Obstetrics and Gynaecology, University of Cagliari, SS 554 – bivio Sestu, Monserrato, 09032 Cagliari, Italy; 2 Queen Margaret University, Institute for Global Health and Development, Edinburgh, EH21 6UU Scotland, UK; 3 Catholic University of Sacred Heart, 00168 Rome, Italy; 4 Health Services Department, Copperbelt University, Kitwe, Zambia; 5 Legal Department “Luca Santa Maria e associati”, Via G. Serbelloni 1, 20122 Milan, Italy; 6 Department of Economics, University of Bologna, Piazza Antonio Scaravilli 2, 40126 Bologna, Italy; 7 Fondazione Policlinico Universitario Agostino Gemelli, IRCCS, Largo Agostino Gemelli 8, 00168 Rome, Italy

**Keywords:** Caesarean section, Obstetrics, Childbirth overmedicalisation, Health Systems of West, Italy, Europe, Cesárea, Obstetricia, Sobremedicalización del parto, Sistemas de Salud Occidentales, Italia, Europa, Kaiserschnitt, Geburtshilfe, Hyper-Medizierung bei der Geburt, Gesundheitssysteme des Westens, Italien, Europa

## Abstract

**Background:**

Improvements in medical technologies have seen over-medicalization of childbirth. Caesarean section (CS) is a lifesaving procedure proven effective in reducing maternal and perinatal mortality across the globe. However, as with any medical procedure, the CS intrinsically carries some risk to its beneficiaries. In recent years, CS rates have risen alarmingly in high-income countries. Many exceeding the World Health Organisation (WHO) recommendation of a 10 to 15% annual CS rate. While this situation poses an increased risk to women and their children, it also represents an excess human and financial burden on health systems. Therefore, from a health system perspective this study systematically summarizes existing evidence relevant to the factors driving the phenomenon of increasing CS rates using Italy as a case study.

**Methods:**

Employing the WHO Health System Framework (WHOHSF), this systematic review used the PRISMA guidelines to report findings. PubMed, SCOPUS, MEDLINE, Cochrane Library and Google Scholar databases were searched up until April 1, 2020. Findings were organised through the six dimensions of the WHOHSF framework: service delivery, health workforce, health system information; medical products vaccine and technologies, financing; and leadership and governance.

**Results:**

CS rates in Italy are affected by complex interactions among several stakeholder groups and contextual factors such as the hyper-medicalisation of delivery, differences in policy and practice across units and the national context, issues pertaining to the legal and social environment, and women’s attitudes towards pregnancy and childbirth.

**Conclusion:**

Mitigating the high rates of CS will require a synergistic multi-stakeholder intervention. Specifically, with processes able to attract the official endorsement of policy makers, encourage concensus between regional authorities and local governments and guide the systematic compliance of delivery units with its clinical guidelines.

## Abstracto

**Antecedentes:**

La cesárea (CS) es un procedimiento que salva vidas y que ha demostrado su eficacia en la reducción de la mortalidad materna y perinatal en todo el mundo. Sin embargo, como cualquier procedimiento médico, la cesárea intrínsecamente conlleva cierto riesgo para sus beneficiarias, especialmente para aquellas que clínicamente no la requieren. La OMS recomienda una tasa de 10–15% de CS como estándar de buena práctica obstétrica. Sin embargo, en muchos países desarrollados el número de CS realizado por año continúa aumentando exponencialmente presionando los recursos limitados de los sistemas de salud. Si bien esta situación expone a las mujeres y a sus fetos a un mayor riesgo quirúrgico, también representa una excesiva carga financiera para los sistemas de salud.

**Tema:**

Utilizando el Framework del Sistema de Salud de la OMS (WHOHSF) y utilizando a Italia como referencia, esta revisión tiene como objetivo resaltar las circunstancias que determinan la excesiva tasa de CS observada en muchos países desarrollados. Analizamos el problema a través de las seis dimensiones del WHOHSF: prestación de servicios, personal sanitario, información del sistema de salud; productos médicos, vacunas y tecnologías, financiación; y liderazgo y gobernanza. Las tasas de CS en Italia se ven afectadas por una compleja interacción entre varios grupos de interés y factores como la ‘hiper-medicalización’ de la prestación, las diferencias en las políticas y prácticas entre las unidades y en el contexto nacional, cuestiones relativas al entorno legal y social junto con las actitudes de las mujeres hacia el embarazo y el parto.

**Conclusión:**

La reducción de las altas tasas de CS requerirá una intervención sinérgica que incluya a las partes interesadas en todos los niveles. Específicamente, intervenciones capaces de atraer el respaldo oficial de los responsables políticos, una alineación de puntos de vista entre las autoridades regionales y los gobiernos locales, y un cumplimiento sistemático de las directrices clínicas por parte de las unidades de prestación de servicios.

## Abstrakt

**Hintergrund:**

Der Kaiserschnitt (CS) ist ein lebensrettendes Verfahren, das sich als wirksam erwiesen hat, um die mütterliche und perinatale Sterblichkeit weltweit zu reduzieren. Wie bei jedem medizinischen Verfahren birgt die CS jedoch ein gewisses Risiko für die Betroffenen, insbesondere für diejenigen, die sie medizinisch nicht benötigen. Die WHO empfiehlt eine Rate von 10–15% CS als Standard für gute geburtshilfliche Praxis. In vielen entwickelten Ländern steigt die Zahl der jährlich durchgeführten CS jedoch weiterhin exponentiell an und setzt die begrenzten Ressourcen der Gesundheitssysteme unter Druck. Während diese Situation Frauen und ihre ungeborenen Babys einem erhöhten Operationsrisiko aussetzt, stellt sie auch eine übermäßige finanzielle Belastung für die Gesundheitssysteme dar.

**Main body:**

Unter Anwendung des WHO Health System Framework (WHOHSF) und am Beispiel Italiens soll diese Übersicht die Umstände aufzeigen, die der in vielen Industrieländern beobachteten übermäßigen CS-Anzahl zugrunde liegen. Wir analysieren das Problem anhand der sechs Dimensionen des WHOHSF: Leistungserbringung, Gesundheitspersonal, Informationen über das Gesundheitssystem; medizinische Produkte, Impfstoffe und Technologien, Finanzierung sowie Führung und Kontrolle. In Italien werden die CS-Raten durch ein komplexes Zusammenspiel verschiedener Interessengruppen und Faktoren beeinflusst, wie z.B. die “Hyper-Medizinalisierung” der Entbindung, Abweichungen in Theroie und Praxis zwischen den Abteilungen und im nationalen Kontext, Fragen des rechtlichen und sozialen Umfelds sowie die Einstellung der Frauen zu Schwangerschaft und Geburt.

**Fazit:**

Um die hohen Raten der CS zu verringern, ist ein synergetisches Vorgehen unter Einbeziehung der Beteiligten auf allen Ebenen erforderlich. Insbesondere sind Interventionen erforderlich, die die offizielle Zustimmung der politischen Entscheidungsträger finden, eine Abstimmung der Standpunkte zwischen regionalen und lokalen Behörden und eine systematische Einhaltung der klinischen Leitlinien durch die Geburtenstationen.

## Background

Childbirth, as pregnancy, is a natural event. However, there is a growing international trend transforming delivery into a surgical procedure mainly via caesarean section (CS) [1]. CS can be a life-saving procedure in circumstances where the life of the mother and her newborn is at risk. However, for a non-complicated labour, conduct of a CS poses unnecessary risks and it places a strain of burden on the entire health system. Clinically, evidence suggests that CS is associated with short and long-term complications, which can affect not only the present delivery but also subsequent pregnancies. Furthermore, CS is associated with longer recovery time (in comparison to vaginal birth (VB) and severe complications which may increase the risk of severe perinatal morbidity and mortality. For the mother, the risk of death is between three to five times compared to VB and increases the risk of severe maternal morbidity by 10 and 15 times [2–5]. In response to the risks and costs which comes with CS, in 1985, the World Health Health Organisation defined the optimal rate of CS to be 10 to 15% of the total number of deliveries per year [6]. This rate was established using the best evidence available at that time and is still considered a standard obstetric target [4]. However, despite this recommendation and the risks attributable to CS, the annual global CS rate has continued to rise at an alarming pace particularly in high and middle-income countries [1, 7, 8]. With an annual average CS rate of 35%, Italy is considered as one of the countries with the highest CS rates in Europe and the world at large. Comparative to Europe which has an average of 24.8%, the CS rates in Italy has gained attention and raised concern [5]. From just 11% in 1980 to 38% in 2011, the pace of CS increase has also raised critical questions about its appropriateness with some scholars suggesting that these procedures are being performed without a clear medical indication [5, 9]. In a recent report by the Italian Ministry of Health based on patient discharge records, the proportion of unnecessary CSs performed in 2010 was estimated to have reached approximately 43%, classifying CS as a national public health threat [10]. As studies have continually shown that part of this situation arises from health system factors, this review aims to summarize and analyse these factors using the globally accepted WHO Building Blocks Health Systems Framework (WHOHSF).

## Methods

### Conceptual framework

The aim of this paper is to contribute to the contextual understanding of the increasing number of CSs being performned in in Italy from a health system perspective. Searching findings of published and unpublished literature, this study summarize the evidence in the context of Italian Health System using the WHOHSF [11]. This framework allows a systematic evaluation of the challenges faced by the HS with emphasis on every component of the system. It has been previously used in many settings especially in low and middle income countries [11–18].

Compared to other studies that have analyzed increasing CS rates from the clinical and health user perspectives [19–21], the WHOHSF was chosen to evaluate the drivers of HS challenges as a series of interacting dimensions, rather than as isolated factors [12, 22].

As demonstrated in this paper, the WHOHSF can be employed to assess not only the appropriateness of health services in general, but also particular practices such as CS delivery. Employing the WHOHSF framework therein offers a full comprehension of its subject based on the key multilevel components of the health system. Furthermore, although previous studies have analyzed the demand-related factors of CS rates in Italy, supply-related issues have remained understudied. For this reason, this systematic review analyzes the situation through all six components of the HS which includes service delivery, the health workforce, health system information, medical products vaccine and technologies, financing, and leadership and governance [11].

### Search strategy

Using the WHOHSF as a guide, we conducted a review of the literature including technical reports, in regard to CS and the six building blocks of National Health Systems (NHS). The Preferred Reporting Items for Systematic Reviews and Meta-Analyses (PRISMA) [23] guided the review strategy. Publications included in this review have been produced by the WHO, the OECD, Italian Ministry of Health and Justice, Italian National Institute of Statistics and the Italian Penal Code. PubMed, SCOPUS, MEDLINE, Cochrane Library and Google Scholar databases were consulted, each search grouping keywords in English and Italian and using Medical Subject Headings [MeSH] related to CS use (i.e. “Caesarean Section”, “Rates”, “Incidence”, “Prevalence”, “Italy”, “Europe”, “Drivers”, “Appropriateness”, “Unnecessary”, “Operative Delivery”, “Surgical Delivery”, “Health System”, “Health Care”, “Regional”, “Disparities”, “Classification”, “Education”, “Policy”, “Practice”, “Medical Liability”, “Incentives”). Further articles were retrieved by screening the reference lists of identified literature. We included manuscripts in Italian and English only. The abstracts of all articles identified in each search were screened and selected for in-depth review when recognised for their relevance to the topic.

### Inclusion and exclusion criteria

A retrospective search was conducted from January 1, 1987 to April 1, 2020. The inclusion criteria for review were:

1. Paper reports evidences pertaining to CS or childbirth on any of the components of the health system as defined by the WHOHSF framework. The primary findings of the study was based on Italy, however to buttress our points, we draw evidences from other similar settings (i.e. Europe and the United States (US).
2. Paper produced in either the English or Italian language.

The exclusion criteria for review includes:

1. Papers not meeting the criteria for inclusion.

### Methodological quality assessment and data extraction

The quality assessment of studies were undertaken using the 8-item Crowe Critical Appraisal Tool (CCAT) (Table 1) [75]. The sections of each document assessed included; the abstract, introduction (background and objective), study design, sampling methodology (when applicable), data collection, ethical considerations (when applicable), and results and interpretation (also classified as discussions). Each section was scored out of five with a possible total of 40. From this, a percentage was calculated and classifying each paper into three levels of evidence quality. Low evidence was classified as literature with a score of < 33.3%, medium quality with a score between 33.3 66.6%, and high quality with > 66.7%. Each paper was assigned a unique identifier including descriptive information such as the study type. Data was independently extracted by VLL, MN and ENO, with each assessment result then compared. In circumstances of disagreement, discussions were held until consensus was reached.

**Table 1:** Quality Assessment Results of all studies included in systematic review

Study	Type of study	1. Preliminaries	2. Introduction	3. Design	4. Sampling	5. Data Collection	6. Ethical matters	7. Results	8. Discussion	9. Total score (/40) and %	Quality of evidence
		Title - Abstract - Text	Background - Objective	Research design – Exposure Outcome - Bias	Size – Protocol - Method	Method - Protocol	Participant ethics - Researcher ethics	Analysis, integration – Essential analysis - Outcome	Interpretation - Generalisation - Concluding remarks		
Villar al. 2007 [2]	Quantitative: prospective cohort	5	3	5	4	5	5	5	5	36 (90%)	High
Dip.Aff.Reg. Autonomie, 2010 [3]	Technical report	na	na	na	na	na	na	na	na	na	Low
Betrán al. 2016 [4]	Quantitative: retrospective survey	5	5	3	5	5	5	4	5	37 (93%)	High
Min Salute, 2016 [9]	Guideline	5	5	4	4	4	3	5	5	35 (88%)	High
Min Salute, 2013 [10]	Institutional report	na	na	na	na	na	na	na	na	na	Low
OECD, 2017 [22]	Technical report	3	5	na	na	3	na	na	3	na	Low
Betran al. 2018 [24]	Qualitative review	5	5	3	4	4	3	4	4	32 (80%)	High
Johanson al. 2002 [25]	Opinion paper	3	3	na	na	3	na	na	3	na	Low
Francese al.2014 [26]	Mixed Methods	4	5	5	3	4	2	4	5	32 (80%)	High
Macones al.2009 [27]	Guideline	5	5	4	4	4	3	4	5	34 (85%)	High
Paterno al. 2016 [28]	Scoping review	5	5	4	4	5	4	3	5	35 (88%)	High
Alfirevic al.2017 [29]	Systematic review	5	5	4	4	5	3	5	5	36 (90%)	High
Sartwelle al.2019 [30]	Opinion paper	3	3	na	na	3	na	na	3	na	Low
Signorelli al.1995 [31]	Quantitative: observational	5	4	5	5	4	3	4	4	34 (85%)	High
Min Salute 2018[32]	Epidemiological report	5	na	na	na	3	na	na	3	na	Low
ISTAT, 2017 [33]	National survey	5	4	5	4	5	na	na	na	na	Low
WHO, 2018 [34]	Guideline	5	5	5	5	5	4	4	5	38 (95%)	High
Fortino al. 2002 [35]	Report	3	na	na	na	na	na	na	3	na	Low
Davoli al. 2016 [36]	Editorial	3	3	3	na	2	na	na	3	na	Low
Ferrè al. 2014 [37]	Unsystematic review	5	4	n.a.	n.a	n.a	n.a	n.a	n.a	n.a	Low
Colais al. 2012 [21]	Quantitative: retrospective cohort	5	5	5	2	4	5	4	5	35 (88%)	High
Piacenza al.2014 [38]	Quantitative: retrospective economic data analysis	4	5	4	2	4	1	4	4	28 (70%)	High
Viselli al. 2014 [39]	Opinion paper	2	2	n.a.	n.a.	2	n.a.	n.a.	2	n.a.	Low
Fusco 2010 [40]	Editorial	3	4	4	4	3	1	2	3	24 (60%)	Medium
Mancuso al.2006 [41]	Quantitative: prospective survey	3	4	3	4	4	2	4	4	28 (70%)	High
Torloni al. 2013 [42]	Quantitative: cross sectional survey	5	4	3	3	4	4	5	4	32 (80%)	High
Clarke al. 2015 [43]	Study protocol for Randomised controlled trial (RCT)	4	4	4	4	5	5	3	3	32 (80%)	High
Clarke al. 2020 [44]	Quantutative: RCT	5	5	4	4	4	3	5	5	35 (88%)	High
AOGOI 2014 [45]	Position paper from the AOGOI society	n.a.	n.a.	n.a.	n.a.	n.a.	n.a.	n.a.	n.a.	n.a.	Low
Camera Deputati 2008 [46]	Parliamentary report	n.a.	n.a.	n.a.	n.a.	n.a.	n.a.	n.a.	n.a.	n.a.	Low
Bilancetti al.2013 [47]	Law book	n.a.	n.a.	n.a	n.a	n.a	n.a	n.a	n.a	n.a	Low
Italian Penal Code, 2017 [48]	Italian criminal code	n.a	n.a	n.a	n.a	n.a	n.a	n.a	n.a	n.a	Low
Eusebi 2011 [49]	Legal research article	n.a	n.a	n.a	n.a	n.a	n.a	n.a	n.a	n.a	Low
Di Landro 2009 [50]	Law book	n.a	n.a	n.a	n.a	n.a	n.a	n.a	n.a	n.a	Low
Bartoli 2018 [51]	Legal research article	n.a	n.a	n.a	n.a	n.a	n.a	n.a	n.a	n.a	Low
Italian Parliament 2012 [52]	Italian law	n.a	n.a	n.a	n.a	n.a	n.a	n.a	n.a	n.a	Low
Italian Parliament 2017 [53]	Italian law	n.a	n.a	n.a	n.a	n.a	n.a	n.a	n.a	n.a	Low
Piras 2016 [54]	Legal research article	n.a	n.a	n.a	n.a	n.a	n.a	n.a	n.a	n.a	Low
Blaiotta 2018 [55]	Legal research article	n.a	n.a	n.a	n.a	n.a	n.a	n.a	n.a	n.a	Low
Traina 2009 [56]	Unsystematic review	4	4	3	n.a	n.a	n.a	n.a	n.a	n.a	Low
Localio 1993 [57]	Quantitative: observational	4	5	5	4	4	2	4	5	33 (83%)	High
Min Giustizia, 2018 [58]	Database on criminal justice in Italy	n.a.	n.a.	n.a.	n.a.	n.a.	n.a.	n.a.	n.a.	n.a.	Low
Forti al. 2010 [59]	Law book	5	5	n.a.	n.a.	n.a.	n.a.	n.a.	n.a.	n.a.	Low
Jena al. 2015 [60]	Quantitative: retrospective cohort	5	5	5	5	4	5	5	5	39 (98%)	High
Forleo al. 2007 [61]	Editorial	3	4	n.a.	n.a.	n.a.	n.a.	n.a.	n.a.	n.a.	Low
Caughey al. 2014 [62]	Guideline	5	5	4	3	4	4	5	5	35 (88%)	High
Fantini al. 2006 [63]	Quantitative: observational	5	4	5	4	4	3	4	5	34 (85%)	High
Robson al. 2013 [64]	Qualitative analysis of published data	4	5	n.a.	n.a.	n.a.	n.a.	n.a.	n.a.	n.a.	Low
Torloni al. 2011 [65]	Systematic review	5	5	5	5	5	3	4	5	37 (93%)	High
Robson al. 2001 [66]	Other (Classification paper and critical review)	2	5	3	3	3	1	2	5	24 (60%)	Medium
Stano al. 1987 [67]	Unsystematic review	n.a.	n.a.	n.a.	n.a.	n.a.	n.a.	n.a.	n.a.	n.a.	Low
Johnson al. 2014 [68]	Unsystematic review	n.a.	n.a.	n.a.	n.a.	n.a.	n.a.	n.a.	n.a.	n.a.	Low
Wee al. 2005 [69]	Guidelines	3	4	3	3	3	2	3	4	25 (63%)	Medium
Velleca al. 2013 [70]	Quantitative: retrospective cohort	4	4	3	4	3	3	3	4	28 (70%)	High
Cavalieri al. 2014 [71]	Quantitative: retrospective cohort	3	5	5	3	3	2	5	2	28 (70%)	High
Contandrio- poulos al.2013 [72]	Quantitative: economic data analysis	2	4	3	2	4	1	3	2	21 (53%)	Medium
Gruber al. 1996 [73]	Quantitative: retrospective cohort	4	5	4	3	4	2	4	4	30 (75%)	High
Tranquilli al.2004 [74]	Brief communication	2	3	2	1	2	n.a.	2	2	14 (35%)	Medium

### Synthesis of results

Results were presented narratively under the components of a health system as provided by the WHOHSF conceptual framework. No meta-analysis was attempted for each section, as the included literature addressed different outcomes.

### Ethical approval

No ethical approval was requested for this study as all evidence used in this review are publicly available.

## Results

### Characteristics of studies

**Fig. 1:**
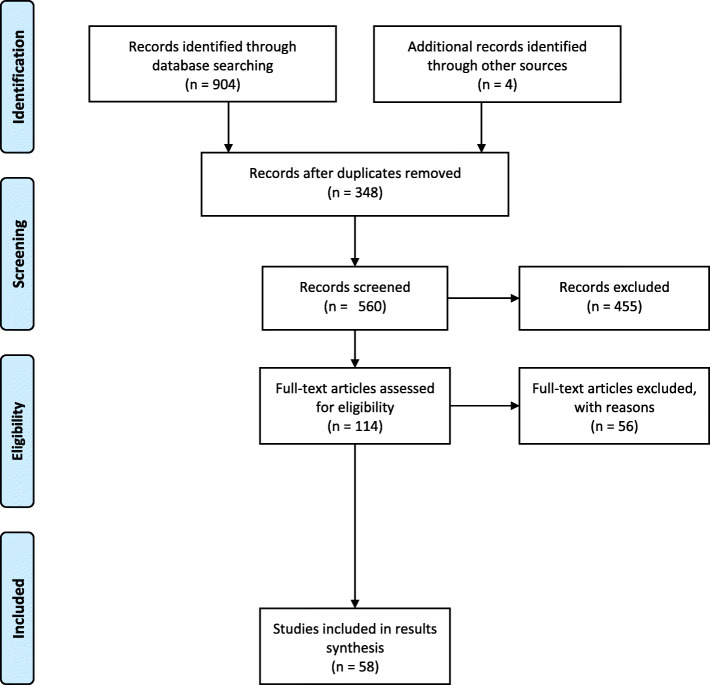
PRISMA Chart describing studies inclusion and exclusion

Figure 1 is a schematic overview of all studies included in this review, with details relevant to each paper outlined in Table 1. One hundred fourteen full text articles were first reviewed and assessed for eligibility, 56 (49%) excluded from the review. Reasons for exclusion included unrelated subject (32/56), academic correspondences (8/56), preprints without peer review (8/56) and articles in languages other than Italian and English (10/56). From the classifications conducted using the quality assessment tool, 41% of the studies were classified as high quality evidence, 9% as medium quality and 50% as low quality. Most of the included studies (71%) were conducted after 2010 with the latest having been published in 2020.

#### Service delivery

**Fig. 2:**
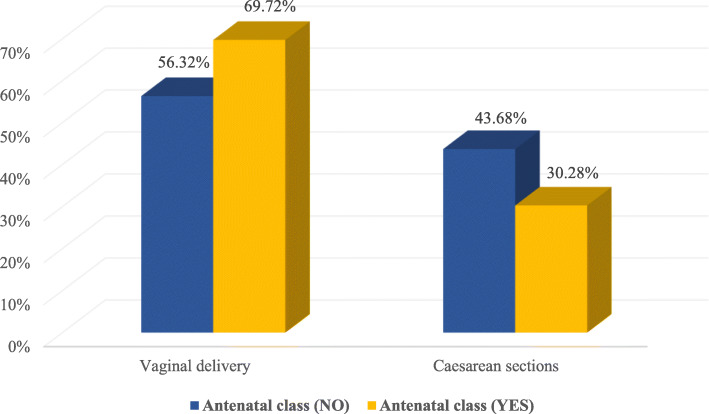
Attendance to antenatal classes and type of delivery (VD and CS) [33]

In recent years, the development of technologies for the monitoring and assistance of deliveries has led to a ‘hyper-medicalization’ of pregnancy and labor [24]. Some of these practices, now a part of the modern obstetric routine, may contribute to the likelihood of CS use. For example, as most deliveries occur in hospital rather than at home, the routine use of technologies to monitor perinatal wellbeing during pregnancy and labor (e.g. electronic fetal monitoring [EFM]) may lead to false-positive diagnoses of fetal distress. Additional contributors to CS also include the increased use of labor-inducing drugs and the use of loco-regional anesthesia [25, 26]. In the case of EFM, recent studies (including a meta-analysis of randomized trials) demonstrate that while continuous EFM in low-risk labor does not improve fetal outcomes, it does significantly increases the rate of CS and vaginal operative delivery [27–30]. Furthermore, while antenatal classes and educational programmes positively correlate to reduced chances to undergo a CS as the mode of delivery (Fig. 2), when compared to other European countries, attendance in Italy is low [33, 34]. Yet the number of antenatal visits and ultrasound examinations performed often exceeds European standards [31, 32]. Although there is no doubt that some of these factors have contributed to making CS deliveries safer than in the past, they have also recast the natural physiological event of childbirth as a medical procedure in which health professionals are highly involved [25, 26].

Studies undertaken across Italy have reported significant geographic variability in the provision and quality of healthcare services, particularly between the northern and southern regions of the country [35, 36]. Closing this gap is one of the main challenges currently facing the national HS [37]. Factors indicative of this service gap include the lower CS rates observed in the North, where the quality and quantity of services provided are generally higher compared to the South. In southern regions, affected by greater financial deficits, CS rates are reported between 60 and 50% [3, 9, 21, 38]. A phenomenon only partially explained by the decentralization of the HS.

**Fig. 3:**
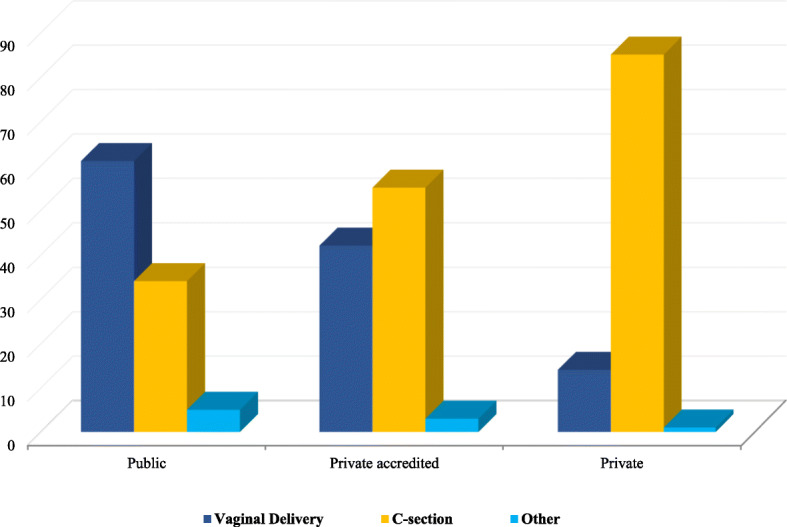
Distribution of all deliveries by type of facility (public, private-accredited, private) [32]

**Fig. 4:**
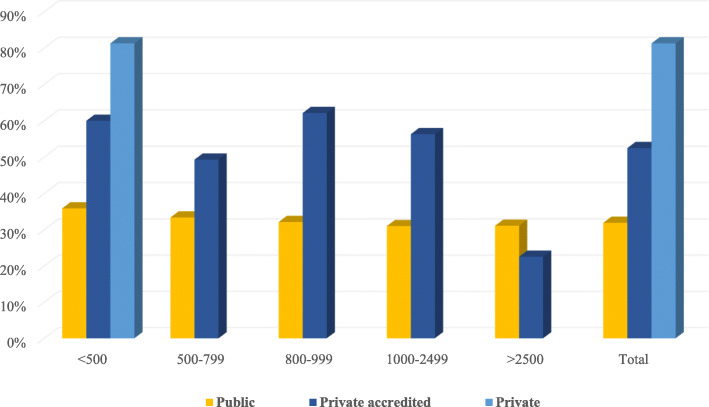
Percentages of CS performed on total of deliveries by level of hospital activity [32]

Significant differences in annual CS rates can also be observed when comparing public and private institutions, and across different delivery units. For example, the number of CS performed is higher in private/private-accredited hospitals compared to public hospitals (53.6% vs. 32.6%) and in locations where the number of deliveries is low [32, 35]. Surprisingly, in hospitals with less than 500 deliveries (often decentralized in rural and semi-urban areas), the number of CSs performed largely exceeds those performed in hospitals with a higher level of activity and where there are more numerous complicated pregnancies and referrals [3, 9] (Figs. 3 and 4). Reasons for this trend might include the profitability of CS, convenience, time management issues, a lack of appropriate services, and staff shortages [3, 36]. The likelihood of performing a CS is also considered to be higher in deliveries that involve staff with less experience, limited assistance, or insufficient equipment; benefitting staff with the ability to schedule a CS delivery and reduce the risk of a complicated VD [39]. Simlarly, additional evidence finds that CSs are less frequent during weekends compared to working days, with the maximum number of CSs performed on Monday and the minimum on Saturday [40].

#### Health workforce

Another factor that may influence provider decisions to perform CS is their perceptions and behavior regarding professional risk [22, 26]. In Italy, healthcare professionals (HCPs) face an average of 15,000 medical liability actions per year. The highest number filed in Europe to date. Among them, obstetricians and gynecologists account for a high proportion of all malpractice suits (approximately 10% [45]). Although more than 90% of all malpractice suits result in acquittal, the personal and professional burden of this risk is noteworthy [46]. Unlike other European countries, medical malpractice in Italy can result in civil and criminal liability [47]. A doctor who is responsible for a patient’s injury or death can face severe punishment, including up to 5 years imprisonment [37, 45, 46, 48, 49]. Additionally, in the Italian civil law system guilt is determined through the case-specific application of legal concepts. Their interpretation left to the discretion of the judiciary. In comparison, in the common law system (used in the United Kingdom, the US and Canada), trials are judged on the precedents of prior cases. Physicians tried under this system only liable for monetary compensation in the event of a conviction [50]. However, under the Italian civil system, doctors practice in conditions of legal uncertainty. The consequences of criminal negligence often unclear and inconsistent [51].

In 2012, Regulation 189/2012 (also known as the “Balduzzi” law) attempted to limit the responsibility of healthcare providers. The law states: “…health workers who in carrying out their activities adhere to guidelines and good practices accredited by the scientific community are not liable for criminal negligence.” [52]. Here, criminal liability is linked in straightforward cases of negligence (*culpa levis*) to an HCPs’ adherence to clinical guidelines and best practices. However, if considered gross negligence (*culpa lata*), the HCP will always be criminally responsible for any damages caused, regardless of their compliance with clinical guidelines [52]. In 2017, the “Gelli-Bianco” amendment aimed to further extend protections for professionals following clinical guidelines [53]. Under the amendment, adherence exempts the provider from criminal suits in cases of demonstrated incompetence/inexperience (“imperizia”). Whereas negligence (“negligenza”) and imprudence (“imprudenza”) remain a criminal offence [54]. Furthermore, this law extends medical liability from the physician alone to include all implicated HCPs (Italian Law No. 8 March 2017). However, despite these advancements, medical practices in Italy still face high levels of litigation, evidenced by inconsistencies in recent malpractice judegments passed by the Court of Cassation [39, 55].As a consequence of this legal uncertainty, physicians’ behavior has progressively changed over time. Some suggest that the fear of legal action has contributed to the practice of ‘defensive medicine’ [53, 60]. This includes protective clinical strategies such as the overuse of diagnostic testing and procedures to minimize legal claims [22, 56, 57].

We argue that for obstetricians and gynecologists, the fear of litigation (particularly in the face of criminal liability) plays a significant role in the high CS rate in Italy [45]. In addition to an ambiguous legal system, another two factors influencing professional perceptions and behavior should be taken into account:

- The history of criminal investigation is highly stigmatizing for healthcare providers, leading the public to question a professional’s reputation despite the outcome of a prosecution.
- The average duration of a criminal trial in Italy, as estimated by the Ministry of Justice in 2016, is approximately 4 years. This excludes preliminary investigations, which can last several years; and cases taken up by the Court of Cassation. This often results in physicians spending a considerable portion of their personal and professional lives under investigation [51, 58].

Additional data collected by the General Medical Council in Rome (*Ordine Provinciale dei Medici-Chirurghi di Roma*) and by researchers from the “*Centro Studi Federico Stella*” at the Catholic University of Milan further detail the magnitude of the problem [59]. Their findings include:

- 78.2% of physicians believe that they are at greater risk of criminal proceedings now than compared to the past
- 65.4% of Italian doctors report undue pressure on their daily clinical practice due to their vulnerability to legal action
- Incentives to practice defensive medicine include the previous judgments of the Court of Justice (57.9%), the experiences of other colleagues (48.4%), and fear of compromising their career (27.8%)
- 77.2% of the doctors interviewed believe the rules governing professional liability negatively affect the quality of care

Encouraged by legal climate in Italy, the practice of defensive medicine may have a significant influence on the quality of healthcare delivery and expenditure (e.g. in the prescription of unnecessary exams and procedures). Further evidenced in the experiences of obstetricians, Francese et al. [26] confirms a correlation between the fear of litigation and obstetricians’ inclination to opt for CS. Particularly in ambiguous cases. In support of this fear, the US-based study by Jena et al. [60] shows a lower rate of malpractice lawsuits against physicians who performed more CSs.

Another workforce element found potentially influencing the high CS rate in Italy, concerns the level of skill required for the effective management of labour and delivery. Further supporting the practice of defensive medicine, obstetrics presents a challenging discipline that requires great experience at the risk of maternal and perinatal death. As part of postgraduate training, obstetricians must be able to predict labor risks, make an assessment and effectively intervene when necessary. Due to this pressure and with the increasing numbers of malpractice claims, countries like Italy and the US have seen more student gynacologists choosing to transfer from obstetrics to train in other “low risk” gynecological areas [61] so that the American College of Obstetricians and Gynecologists (ACOG) submitted a call for US-based gynaecologists not abandon the obstetrics discipline too soon [62]. Although there is a lack of similar evidence analyzing the training decisions of student gynaecologists in Italy, the Italian Association of Hospital Gynecologists and Obstetrics (AOGOI) regularly reports a dwindling interest in the uptake of obstetrics at the post-graduate level [61]. This “educational insecurity” could therefore influence the decisions of young doctors, especially in cases where the decision to perform a CS is percieved to be easier than managing an unpredictable VB.

#### Information

In Italy, the National Outcomes Programme (Piano Nazionale Esiti – PNE) includes CS rates as one of its indicators of quality of obstetric care at the inter-hospital and international level [21, 40, 63]. Lower CS rates per annum considered to indicate a higher quality of obstetric practice and better hospital performance, ultimately influencing a hospital’s national ranking [63]. In particular, ‘primary CS’ (i.e. a CS performed for a woman for the first time) represents one of the most important quality indicators informing national healthcare development and intervention [36, 40]. As endorsed by the WHO, this monitoring of CS rates at facility-level is considered essential to guaranteeing that the procedure is being offered to the women who need it [4]. At present, there is no internationally approved classification for CSs [4]. Therefore, the absence of a standardized tool to classify CSs remains an obstacle to understanding trends in its delivery, preventing health facilities from comparing and monitoring CS rates. This being cited as one of the key barriers to the reduction of CS rates [4, 64].

To encourage the adoption of a common tool, in 2011 the WHO carried out a systematic review of the different methods being used to classify CSs around the world. As a result, the WHO agreed that the Robson 10-Group Classification System (RTGCS) was the most comprehensive and appropriate tool available [65]. Based on five clinical characteristics, the RTGCS groups all women into 10 classes, which are mutually exclusive and fully comprehensive [66]. This allows researchers to draw comparisons both at the facility and population levels. Data available from the RTGCs are potentially beneficial to the establishement of a behavioral protocol and contemporary standards for CS [32]. However, a number of hospitals and delivery units (especially small/private institutions) have not yet adopted the RTGCS.

#### Medical products, vaccines and technologies

The ‘medicalization’ of childbirth has created an environment in which medical products and technologies play an essential role in the delivery process now more than ever. In this paper we have discussed the impact of this medicalization on the likelihood of CS delivery. In addition to this phenomenon, the theory of induced-demand would suggest that after the supply of a good increases, more of that good is consumed [67, 68]. As with all surgical procedures, CSs require a wider use of medical products and technologies compared to VDs, such as anesthesia, and other specialized products and equipment including the use of postpartum drugs (i.e. antibiotics, anticoagulants and pain-killers) [69]. For some of these drugs, their use is recommended for as long as a month after delivery. Furthermore, a study conducted by Villar et al. [2] on a cohort of approximately 100,000 women showed how those who underwent a CS were 5 times more likely to require antibiotic treatment compared to women who delivered spontaneously. Following this logic, it may be argued that the continuous availability of the tools of CSs may also play a role in decision-making relevant to delivery modes. This supply encouraging clinicians to perform CSs, particularly in ambiguous situations.

#### Financing

The financing mechanism of the Italian HS constitutes a national health insurance program (i.e. universal health coverage intervention), providing individuals access to health care, free at the point of need. Constitutionally, all residents are entitled to receive a package of services, the “*livelli essenziali di assistenza*” (LEA) (essential levels of care). This includes most maternal health services, such as antenatal care, delivery, and postnatal care. The LEA determined and ensured by the national government. However, since the decentralization of the Italian HS, the LEA has been delivered both in publicly and privately accredited hospitals by the Regional Health Service (RHS) and local health authorities [37, 70]. Under this organisation, since 1995 reimbursement schemes have been based on DRGs (diagnosis-related groups). A system that classifies patients discharged by NHS hospitals in homogenous reimbursement groups [70]. Here, a CS is generally reimbursed at a higher value (€ 2092/2782, per procedure with/without complications) than a VD (€ 1272/1619^,^ per procedure with/without complications) [10]. Reimbursements for deliveries are also becoming highly varied across different RHSs. Therefore, it is unsurprising that where RHS tariff systems provide a higher financial difference, providers demonstrate a higher rate of medically unjustifiable CSs. Cavalieri et al. [71] ascribes these findings to the theory of induced demand, suggesting that the inappropriate use of CS is a direct result of financial incentives. Thus, those RHSs receiving high reimbursement for CSs retaining the difference in funds [26, 32, 71]. Although the influence of this incentive varies across providers, it is evident that it is the case of private hospitals and facilities with a small number of deliveries. Such facilities, despite attending to lower numbers of high-risk pregnancies, conduct high CS rate [71].

Additionally, the income-target hypothesis conceptualises that money influences work practices in similar ways to the phenomenon described above. Individuals and institutions prone to adjustments in practice that would accomplish a high income [72]. Therefore, intersecting factors such as the increased reimbursement of CSs and decreasing fertility rates in Italy may also infleunce physicians’ decisions concerning CS deliveries [73]. The recommendation of CS is cited in the literature as an example of standard care practices for the achievement of a ‘target income’. This implies that physicians/institutions may use their position and authority over their patients, to generate income [73].

#### Leadership/governance

In Italy, all medical procedures including CSs are guided by the Ministry of Health (MOH) national guidelines. The objective of these guidelines is to explicitly clarify the clinical circumstances in which a CS should be performed [9]. However, it has been observed (particularly in the South), that providers do not always adhere to MOH recommendations [21]. Thus, according to the guidelines many continue to perform ‘inappropriate’ CSs [3]. Reasons for this disregard may include financial gains or organizational constraints; these factors enhanced by the decentralization of the HS where each region has a certain degree of fiscal devolution. For example, in Campania (the Italian region with the highest CS rate, at approimatley 60%) the number of CSs performed has risen in parallel with the introduction of the DRG reimbursement scheme [39]. This situation is also accentuated by the fact that some women perceive CS to be beneficial to them and their babies, and are allowed to request the procedure even in absence of clinical indicators supporting the principle of “auto-determination” [46, 74]. However, at present there is no specific law regulating elective CSs in Italy. The decision to offer this service left to the physician’s discretion [9].This may also contributing to increasing CS rates, especially when women are not accurately informed of the potential risks of the procedure [32, 41].

## Discussions

While it is clear from this review that health system factors drives the high CS rate, other demand factors and preference of women is also an influencing factor. Women have become increasingly more involved in the decision-making processes of their doctors throughout their pregnancies. Consequently, women’s preferences for a mode of delivery may also influence service provision. According to Francese et al. [26], women’s cultural perceptions of pregnancy and delivery have also changed. Today, women are less likely to accept the risk of adverse outcomes. Additionally, with more frequency, women perceive CSs as a safer and less painful procedure when compared to VDs [41]. At present, only one study has investigated preferred mode of delivery in Italian women. The main reported reasons for choosing a CS delivery among Italian women was the fear of pain assocated with VD and the opportunity to schedule the delivery. Furthermore, CS is perceived to be safer and less painful for the baby. Also, CS is associated with a reduced delay in returning to sexual activity and reduced odds of sexual impairment for the mother [42]. Women self reported that their choices was first influenced by a gynecologist then family and friends [42]. In such a context, it is important that women are properly informed about the potential risks and benefits of CS. A possible way to achieve this is through the provision of services that promote a positive pregnancy experience by including women in their obstetric care decisions from the time of conception to delivery. Examples of these services reported in the literature include ad hoc meetings, informative material and online services. Such interventions are usually designed to be women-centered and have demonstrated to positively influenced women’s decision on the mode of delivery [43]. For example, an RCT conducted in Italy and other European countries in the period 2014–2015 to investigate women’s attitude towards trial of labour after CS found that engaging women in decisions of delivery increased vaginal delivery by 11%. This marginal improvement in vaginal delivery using this strategy, according to the authors, could avert 160,000 CS and the health system €150 milion annually [43, 44]. Furthermore, the high CS rate in Italy warrants a highly accurate monitoring of epidemiological data and hospital activity releant to CSs [21]. While the WHO has strongly advocated for the systematic use of the Robson classification, until now it has not been fully implemented in Italy [4]. This is particularly true in decentralized hospitals and private institutions, which were found to be major contributors to CS rates [9, 35]. Among factors found to pontentially influence CS trend in Italy, this review has allowed to highlight the importance of the legal context in the practice of medicine in Italy, and area that is usually underinvestigated. However, while the influence of fear of litigation on medical decision-making is supported by the literature, more research is necessary to better understand the impact of defensive medicine on care outcomes.

## Conclusions

In this study, we have considered the systemic complexities of increased CSs rates in Italy, emphasizing how different drivers, including non-medical factors, have contributed to current trends. In doing so, we recognize that our novel use of the WHOHSF as a diagnostic tool has made it possible to highlight how high CSs rates in Italy are not only an isolated clinical problem, but also one with roots in all six components of the HS. Consequently, it becomes clear that effective approaches to reduce unnecessary CSs would require coordinated efforts by all stakeholders. Particularly, with the official endorsement and support of policy makers, the alignment of regional authorities with local governments and pratictioners’s increased adherence to clinical guidelines. Furthermore, we find the WHOHSF to be a critical tool for the future assessment of areas commonly neglected by medical journals. This offers the potential to facilitate the implementation of integrated evidence-based interventions, develop an cohesive health assistance model and improve the general quality of sexual and reproductive health services. Ultimately, this will contribute to the much needed optimization of CS use not only in Italy, but also in other high-income countries around the world.

## Data Availability

This article relied on secondary data and published articles. All information/data used are readily available for further analysis via the provided links in the reference section.

## Abbreviations

CeDAP: Certificato di assistenza al parto
CSs: Caesarean sections
DRG: Diagnosis-related groups
EFM: Electronic Fetal Monitoring
NHS: National Health System
HS: Health System
ISTAT: Istituto Nazionale di Statistica
LEA: Livelli Essenziali di Assistenza
MEDLINE: Medical Literature Analysis and Retrieval System Online
MINSAL: Ministero Della Salute
MOH: Ministry of Health
OECD: Organisation for Economic Co-operation and Development
PNE: Piano Nazionale Esiti
RCT: Randomized Controlled Trial
RHS: Regional Health Service
RTGCS: Robson 10-Group Classification System
TOLAC: Trial of Labour After Caesarean delivery
VB: Vaginal Birth
VBAC: Vaginal Birth After Caesarean delivery
VD: Vaginal Delivery
WHO: World Health Organisation
WHOHSF: World Health Organisation Building Blocks Health Systems Framework
